# Case of Malignant Epulis of the Lower Jaw—with Operation by Prof. Freer

**Published:** 1872-07

**Authors:** 


ARTICLE III.—*Case of Malignant Epulis of the Lower Jaw—with Operation by Prof.* FREER. Clinic at Cook County Hospital, May 14, 1872.

(Present: Professors GUNN and POWELL, Drs. PARKES, OWENS, CURTIS, and many other physicians of the city, and the class of medical students.)

(THE OPERATION. Reported by Dr. O'BRIEN.)

Professor Freer said: "The case here presented to you, gentlemen, is an unusual one; unusual for the *magnitude* of the tumor rather than for the nature of it. At first glance, you would naturally suppose that this tumor was an enormous, projecting tongue, but studying it more closely and looking into the mouth attentively, you would discover the tongue *intact* and tilted up into the fauces, where it has been crowded by the rapid enlargement of this malignant tumor of the lower jaw. The bone itself, covered by the growth, has been crowded by it down upon the larynx, where it forms an apparent girdle to the tumor; the lower lip is widely stretched and almost obliterated; the sub-maxillary glands, greatly enlarged by the disease, rest upon the neck on either side of the bone. The patient can scarcely breathe as you observe, on account of the larynx being encroached upon by the disease; he swallows with extreme difficulty, suffers great pain, and will die in a day or two of suffocation, if allowed to remain as he is.

"Four months and a week ago, this case started with a little tumor surrounding two of the molar teeth in the left side of the lower jaw; it was epulis. This disease always begins in the periosteum of the alveolus, and involves the gum secondarily; it is usually simple and non-malignant, but in some cases, as in this, it is enormous in size, and malignant. There can be no question of the malignancy of a tumor presenting such a history and character as the one before us. Paget calls these tumors fibrous, but again in his work he calls them malignant, and describes them as recurring after removal. Erichsen describes them briefly as malignant epulis.

"The patient begs for an operation. We can scarcely hope for more than a little palliation of his sufferings. He is aware of the danger, and says that he prefers to take the risks with the small chance of improvement, to suffering intensely as now. The earnest solicitations of the patient, the certainty of speedy death from suffocation if not relieved, and, on the other hand, the chance of improvement however slight, which may follow a successful operation,—these considerations induce me to give the patient the only chance that is left for him.

"The difficulties are manifold, the most prominent of them being hemorrhage; the tumor is exceedingly vascular, as you might readily suppose, as it has grown rapidly. We must remove the lower jaw; and this involves another danger, namely, suffocation from the dropping of the tongue upon the glottis when its connections are severed—an accident which has occurred in the hands of the ablest surgeons. We must allow no bubble of air to enter the veins. But I approach these dangers with confidence, having such able support and assistance."

Chloroform given, Prof. Freer transfixed the tongue with a tenaculum, which was intrusted to an assistant to prevent the danger of obstruction to the glottis; then with a scalpel he made an incision from the median line around the right half of the lower jaw to its angle and up the ramus, cutting in its course the facial artery, which presented the curious anomaly of bleeding *only* from its distal end—an anomaly which was explained in a few moments;. the second stroke of the knife was around the left jaw, then a perpendicular incision in the median line, formed with the preceding an incised cross over the tumor, with four corners of flaps to be dissected up; dissecting these, first upon the left side, and then the right, Prof. Freer next passed a strong ligature around the left ramus, with a stout blunt curved needle, hugging closely the jaw. To the ligature a chain saw had been attached, it was drawn around the ramus, the handle fastened, and in a few seconds the ramus was divided.

In the artery which bleeds now, we recognize the inferior dental; while assistants are applying to it the actual cautery, Prof. Freer finds time to say, "Do not extend your incisions upon the ramus of the jaw high enough to cut the facial nerves; it is not necessary even when removing the jaw at the articulation."

The right ramus was cut through in the same way, and the rest of the operation can only be described by saying that with knife, blunt spud, and fingers, the surgeon excavated the lower jaw and tumor. In getting out the right sub-maxillary gland, the facial artery was cut again, and *now* bled from the proximal side; it had been *ligated* by the hardened gland; wherein, we saw the explanation of the previous anomaly.

Following every stroke of the operator's knife, his distinguished colleagues promptly met each danger as it arose, and the whole of the formidable operation was completed with the loss of not more than two ounces of blood. After the removal of the tumor, the actual cautery was applied to the tissues and the wound closed by hare-lip sutures.

(HISTORY OF THE CASE. Reported by Dr. MONTGOMERY, House Surgeon.)

John Gill, 50 years of age, laborer, native of Ireland, was admitted into Hospital May 10th, 1872.

The patient is able to talk so as to be but imperfectly understood, but by questioning closely we are able to obtain the following history:

Patient resides in the State of Michigan, and has lived there for a good many years. Says that he has never been sick, but rugged, and thought he could stand almost anything until his present trouble came upon him. A little over four months ago he first noticed a small lump on the left side of the gum of the lower jaw. It was not painful when he first noticed it, but it began to enlarge very soon and extend toward the right side and become painful. In about three weeks almost the whole of the lower gum was affected, and the front teeth were so pushed up that he could pull them out with his fingers. The tumor has rapidly increased in size until this time, when it protrudes from the mouth in a forward and downward direction, pushing the lower lip down as low as the chin. It resembles, closely, a huge tongue with a raw surface protruding from the mouth and pushing the lip down. The tongue is crowded backward, almost closing the glottis. The sub-maxillary and sub-lingual glands are also involved. The pain has increased with the size of the tumor, but is more severe at night; is sharp and stabbing in character. Patient has to take large doses of morphine to secure rest at all. Appetite has been good all the time and is at present, but the patient is only able to take fluid nourishment, and swallows that with difficulty. Has much dyspnœa. Is much emaciated, and apparently almost exhausted.

May 14. It has been decided to try to remove the tumor. Operation performed about 3 P. M. Ten P. M. Pulse 104; respirations 16. Patient has fully reacted from the operation, and is sleeping; injections of beef-tea have been ordered.

May 15—8 A. M. Pulse 120; respirations 16. Is rational, and able to swallow fluids if put into the mouth with a spoon. 9 P. M. Pulse 148; respirations 28. Patient has taken considerable nourishment.

May 16—8 A. M. Pulse 130; respirations 20. Did not sleep much, but shakes the head when asked if he has much pain.

May 17. Pulse 132; respirations 28. Rested quietly but is very weak; will not take nourishment. Died 5 P. M.

(ITS PATHOLOGY. Report of Dr. DANFORTH.)

I find the usual elements of rapidly-growing cancer, namely:

First—Innumerable minute granules.

Secondly—Small nucleated cells, generally with only a single nucleus. These small cells, according to the common idea, would scarcely be taken for those belonging to a malignant growth. They seem to be young cells, in process of formation, and hence, those which present the least departure from the so-called "benignant" type.

Thirdly—Various forms of large cells, conforming to no particular law of type, assuming all varieties of shapes, and evidently growing in all directions in which absence of resistance renders growth possible. Some of these large cells contain a single nucleus, but most of them have several nuclei. Some of them contain oil drops, and are, therefore, undergoing "fatty degeneration"—a form of "retrograde metamorphosis" not uncommon in cancer. Some of them are round, some oblong, some spindle-shaped, some caudate, some angular, in fact, scarcely any two of them are alike, and if I describe any one of them, the description will not apply at all to its next neighbor. Therefore, *because* I cannot describe them; *because* of their lawless mode of growth; *because* of their reckless behavior *during* growth and *after* they are grown; *because* they have cast off their allegiance to all recognized laws of growth (or law of type), they are cancer cells.

Fourthly—Several cylindriform bodies containing many nucleated cells—the "nucleated cylinders" of Koester.

Fifthly—One specimen which I received came from that portion of the growth surrounding the symphysis of the jaw, which, on account of the extreme erosion of the lip, was constantly exposed to the atmosphere. A dense blue mold quickly sprang up upon this part of the specimen, and microscopic section revealed filaments of the parasitic plant penetrating the mass in every direction. As I immediately placed the specimen in a closely covered capsule, and covered it with glycerine and acetic acid, I do not think the germs found access to it after it came into my possession. Nor do I believe that the presence of the plant had any direct relation with the pathology of the case—but that it was purely accidental, the result of constant exposure previous to the operation.

